# The Presence of Anti-HLA Antibodies before and after Allogeneic Hematopoietic Stem Cells Transplantation from HLA-Mismatched Unrelated Donors

**DOI:** 10.1155/2012/539825

**Published:** 2012-10-24

**Authors:** Anna Koclega, Miroslaw Markiewicz, Urszula Siekiera, Alicja Dobrowolska, Mizia Sylwia, Monika Dzierzak-Mietla, Patrycja Zielinska, Malgorzata Sobczyk Kruszelnicka, Andrzej Lange, Slawomira Kyrcz-Krzemien

**Affiliations:** ^1^Department of Hematology and BMT, Medical University of Silesia, Dabrowskiego 25, 40-032 Katowice, Poland; ^2^HLA and Immunogenetics Laboratory, Regional Blood Center, Raciborska 15, 40-074 Katowice, Poland; ^3^Lower Silesian Center for Cellular Transplantation with National Bone Marrow Donor Registry, Grabiszynska 105, 53-439 Wroclaw, Poland

## Abstract

Although anti-human leukocyte antigen antibodies (anti-HLA Abs) are important factors responsible for graft rejection in solid organ transplantation and play a role in post-transfusion complications, their role in allogeneic hematopoietic stem cell transplantation (allo-HSCT) has not been finally defined. Enormous polymorphism of HLA-genes, their immunogenicity and heterogeneity of antibodies, as well as the growing number of allo-HSCTs from partially HLA-mismatched donors, increase the probability that anti-HLA antibodies could be important factors responsible for the treatment outcomes. We have examined the incidence of anti-HLA antibodies in a group of 30 allo-HSCT recipients from HLA-mismatched unrelated donors. Anti-HLA Abs were identified in sera collected before and after allo-HSCT. We have used automated DynaChip assay utilizing microchips bearing purified class I and II HLA antigens for detection of anti-HLA Abs. We have detected anit-HLA antibodies against HLA-A, B, C, DR, DQ and DP, but no donor or recipient-specific anti-HLA Abs were detected in the studied group. The preliminary results indicate that anti-HLA antibodies are present before and after allo-HSCT in HLA-mismatched recipients.

## 1. Introduction

Allogeneic hematopoietic stem cell transplantation (allo-HSCT) is an effective treatment of both congenital and acquired disturbances of hematopoiesis, especially of hematological malignancies.

The selection of the optimal donor is based on high-resolution HLA typing. The MHC (Major Histocompatibility Complex) contains more than 200 genes which are situated on the short arm of chromosome 6 at 6p21.3. It is divided into three main regions: HLA class I (containing *HLA-A*, *B*, and *C* genes), class II (containing *HLA-DR*, *DQ*, and *DP* genes), and class III region. The role of HLA molecules is to present peptides to T cells (both CD4 and CD8 T cells), enabling them to recognize and eliminate “foreign” particles and also to prevent the recognition of “self” as foreign. HLA mismatches may occur at antigenic or allelic level; the first are characterized by amino acid substitutions in both peptide-binding and T-cell recognition regions, whereas the latter are characterized by amino-acid substitution in the peptide binding regions only [1].

HLA antigens are recognized by immunocompetent T cells, what may lead to graft failure, graft versus host disease (GVHD), and other posttransplant complications as well as to favorable graft versus leukemia (GVL) effect. HLA molecules bear multiple antigenic epitopes, many of which are the so-called “public” epitopes that are shared among the products of several different HLA alleles, resulting in the apparent cross-reactive groups of antigens (CREGs). These shared epitopes may be responsible for patient's sensitization to multiple HLA antigens, despite a single antigen mismatch only [2–4].

The participation of cellular arm of immunological response to HLA antigens is well known, but the role of humoral arm of immunity is also very interesting, especially when we consider the enormous polymorphism of HLA-genes, their immunogenicity and huge heterogeneity of antibodies. Antibodies are glycoproteins that belong to the super-family of immunoglobulins [5]. The basic structural units of antibodies are two heavy chains (*α*, *γ*, *ε*, *δ* or *μ*) and two light chains (*κ* or *λ*). The type of a heavy chain determines the class of antibody: IgA, IgG, IgM, IgE or IgM [6]. The region of chromosome that encodes the antibody is large and contains several distinct genes. The locus containing heavy chain genes is found on chromosome 14; loci containing *κ* and *λ* light chain genes are found on chromosomes 2 and 22, respectively. The enormous diversity of antibodies allows the immune system to recognize an equally wide variety of antigens [5]. It has been known that humans produce about 10 billion different antibodies capable of binding a distinct epitope of an antigen [7]. Such a diversity of antibodies is caused by domain variability, recombination, somatic hypermutation and affinity maturation, class switching, and affinity designations [8–10]. Anti-HLA Abs may be present in healthy individuals [11, 12]. The sensitization to MHC antigens may be caused by transfusions, pregnancy, or failed previous grafts [13]. Anti-HLA Abs are more frequently detected in patients with hematological disorders due to their alloimmunization, resulting mainly from common use of transfusions [14].

The clinical significance of anti-HLA Abs is well known in the field of transfusional medicine. The presence of anti-HLA Abs in patients is one of the major causes of platelet transfusion refractoriness [15]. On the other hand, anti-HLA Abs present in blood products have been shown to be a major cause of transfusion-related acute lung injury (TRALI) [16, 17]. The role of anti-HLA Abs is also well known in solid organ transplantation—especially in kidney transplantation, because transplanted kidneys are highly susceptible to antibody-mediated injury [18, 19]. Antibodies produced before kidney transplantation (reacting with donor's HLA antigens) induce hyperacute or acute vascular rejections which frequently result in transplant failure [20, 21].

Despite the well-recognized role of antibody-mediated rejection in solid organ transplantation, the graft rejection following allo-HSCT is generally attributed to cytolitic host-versus-graft (HVG) reaction mediated by host T and NK cells, that survived the conditioning regimen [22–25]. Engraftment failure rate is approximately 4% in allo-HSCT from matched unrelated donor (MUD) and about 20% in cord blood or T-cell-depleted haploidentical transplantations [26, 27]. Antibody-mediated bone marrow failure after allogeneic bone marrow transplantation can be also caused by antibody-dependent cell-mediated cytotoxicity (ADCC), or complement-mediated cytotoxicity [28–30]. In ADCC, the cytotoxic destruction of antibody-coated target cells by host cells is triggered when an antibody bound to the surface of a cell interacts with Fc receptors on NK cells or macrophages. Preformed antibodies present at the time of hematopoietic stem cell infusion are unaffected by standard transplantation conditioning regimens, T- or B-cell immunosuppressive drugs or modulatory strategies given in the pretransplantation period [31].

Albeit the T-cell-mediated cellular immunity is the primary barrier for bone marrow allorejection in nonsensitized recipients in the animal models (mice), the humoral arm of the immune response plays a very important, previously unappreciated, role in the rejection of allogeneic stem cell transplantation in sensitized mice and in such case the rejection of a bone marrow is T-cell independent [31, 32]. Moreover, the achievement of a mixed allogeneic chimerism resulted in reverse of the sensitization in allosensitized recipients [30, 33]. Probably not only antigen-specific but also cross-reactive or broadly reactive alloantibodies may be responsible for the graft failure [32]. Spellman and Bray have demonstrated in a retrospective, case-controlled study that the prevalence of donor-specific anti-HLA antibodies was higher in a group of mismatched unrelated donor recipients who suffered graft rejection than in a control group that engrafted. Among the 37 recipients who failed to engraft 9 (24%) possessed DSAS against HLA-A, B, or DP, but only 1 (1%) recipient of 78 controls did [34]. In the study of Ciurea et al. DSAS was the single most important factor associated with graft failure and HLA-mismatches increased the occurrence of donor-specific HLA antibodies in MUD transplantation [35]. Takanashi et al. demonstrated the impact of anti-HLA antibodies on engraftment after myeloablative single unit cord blood transplantation. Patients with anti-HLA antibodies experienced slower neutrophils and platelet recovery than antibody-free patients. Although no effect of anti-HLA antibodies on GvHD grade II-IV, relapse, or TRM has been observed, the overall and event-free survival were significantly inferior in antibody-positive patients [36]. Similar observations were made after double umbilical cord blood transplantation [37].

As presented above, the influence of anti-HLA Abs, including Abs directed against mismatched antigens, on the results of allo-HSCT, especially on graft failure, has been proved in several reports. However, in patients following allo-HSCT, the series of time remote complications may occur. As antibodies appearing in the result of the earlier immunization are detected before transplantation, the question of their presence and specificity after transplant, after the myeloablative conditioning treatment, and during administration of immunosuppressive therapy is open, when the hematopoietic function is taken over by the donor's cells. The first cells to reconstitute (within the first 100 days) after the transplantation are granulocytes, monocytes, macrophages, and NK cells. In contrast, T and B lymphocytes remain severely reduced and their function is impaired from 6 months to 1 year after the transplantation [1].

Therefore, the aim of our study was to examine the presence and the specificity of anti-HLA antibodies before and following the allo-HSCT.

## 2. Materials and Methods

We included 30 patients who received allo-HSCT from partially HLA-mismatched unrelated donors and who agreed to participate in the study. Donors lacking full HLA compatibility with recipients were chosen when compatible donors were not available. Standard high-resolution allelic typing of HLA-A, B, C, DRB1, and DQB1, without HLA-DP, was performed. The study was carried out in the Department of Hematology and Bone Marrow Transplantation of the Medical University of Silesia in Katowice, Poland, between 2007 and 2011. The examination of patient's sera was scheduled at 4 time points: before the start of conditioning treatment and 30 days, 100 days, and 1 year after transplant.

The preparative treatment was myeloablative in 28 (93%) and reduced in 2 (7%) pts. Standard GVHD prophylaxis consisted of pretransplant antithymocyte globulin, cyclosporine A in initial dose 3 mg/kg i.v. starting from day −1 with dose adjusted to its serum level and shift to oral administration about day +20, methotrexate 15 mg/m^2^ i.v. on day +1, and 10 mg/m^2^ i.v. on days +3 and +6. Methylprednisolone at dose 2 mg/kg i.v. was the first line therapy of aGVHD symptoms; in few patients mycophenolate mofetil or tacrolimus was used. The source of cells was the bone marrow in 9 (30%) and peripheral blood in 21 (70%) patients.

The detailed characteristics of the study population are presented in Table 1.

Patient's sera were tested for the presence of anti-HLA Abs in the HLA and Immunogenetics Laboratory of Regional Blood Center in Katowice, Poland. Anti-HLA A, B, C, DR, DQ, and DP antibodies were detected and identified using the ELISA-based DynaChip Technology. The DynaChip HLA Antibody analysis system utilizes microchips spotted with purified HLA antigens immobilized on the surface of the glass chip. Test serum was free of aggregates and excess lipids before testing. This was achieved by centrifugation for 10 minutes at 10,000 g. The clarified supernatant was diluted with the Sample Diluent contained within the kit and then it was added to the DynaChip wells. Anti-HLA Abs present in the test serum were bound to the HLA antigens on the surface on the chip. Bound antibodies were then detected using the Antibody Detection Reagent (antihuman IgG and horseradish peroxidase complex). The assay was completed with colorimetric detection. The resulting patterns of blue-positive and clear-negative spots were recorded by the software and subsequently automatically analyzed by the DynaChip Analysis Software. The presence of at least one anti-HLA antibody was regarded as presence of anti-HLA Abs, whereas if the examined serum contained antibodies against more than 50 different HLA antigens they were regarded as “anti-HLA Abs to many specificities.” Applied DynaChip HLA Antibody analysis system did not allow to measure the concentration of detected antibodies.

The study has been approved by the responsible Ethical Committee of Medical University of Silesia.

## 3. Results

Anti-HLA Abs were detected in 26 (86.6%) patients. Anti-HLA Abs against HLA class I, II, or both were detected in 8 (26.6%), 2 (6,6%), or 16 (53.3%) patients, respectively. In 4 (13.3%) patients they were detected before transplant only, in 10 (33.3%) patients after transplant only, and in 12 (40%) patients both before and after transplantation. In 4 (13.3%) patients anti-HLA Abs were not detected neither before nor after allo-HSCT. Anti-HLA Abs directed against the class or antigens of mismatched HLA were detected in 4 patients before transplant and in 9 patients after transplant. In 5 patients we identified antibodies with the same specificities before and 30 days after the transplantation (as presented in Table 2, cases' numbers: 19, 20, 21, 23, and 30). Although we did not identify neither donor or recipient allele-specific anti-HLA Abs, antibodies that detected after transplant in 3 patients belonged to the same CREG (Cross-Reactive Group) as recipient's mismatched HLA antigen (as presented in Table 2, cases' numbers: 19-10CREG, 24-5CREG, and 29-12CREG). These antibodies were detected more than 100 days after transplantation, so it is very likely that they were produced by donor cells.

The specificities of anti-HLA Abs detected before allo-HSCT and at different time-points after transplant are presented in Table 2. We have succeed only partially in consequent collecting sera at all scheduled timepoints from patients included into the study for analysis due to the fact that some patients were referred to our center for allo-HSCT from remote parts of Poland. After allo-HSCT they have moved for care to their home centers and collection of the complete set of sera from them was impossible.

## 4. Conclusions

Our preliminary results indicate that preformed anti-HLA Abs can be detected before and may also appear after transplant in mismatched allo-HSCT recipients. Anti-HLA Abs present in 3 patients were directed against HLA antigens which belonged to the same serological Cross Reactive Groups as the mismatched HLA antigens.

In 5 patients anti-HLA Abs directed against the same HLA antigen were detected before and after allo-HSCT what may indicate that they were not destroyed during the myeloablative conditioning treatment and standard immunosuppressive therapy. These antibodies belonged to the same serological Cross Reactive Group as the recipient's but not donor's mismatched HLA antigens, so it is possible to conclude that donor's cells may produce anti-HLA Abs against the recipients cells after allo-HSCT. Therefore, they may theoretically be responsible for induction of several immunological posttransplant complications. Antibodies detected after transplantation may also result from immunization, for example, by transfusions, as allo-HSCT recipients often require intensive supportive treatment with blood derivatives.

We believe that our observations help to better understand the immune mechanisms contributing to allogeneic sensitization which may influence allo-HSCT results. It is possible that sensitized patients who possess anti-HLA antibodies before or after the transplantation could benefit from modification of conditioning and immunosuppressive therapeutic approaches in the future.

Presented preliminary outcomes of 30 patients are based only on part of our whole study group which consists of 70 patients. The statistical analysis aimed to reveal the eventual impact of anti-HLA Abs on allo-HSCT results will be performed after completion and examination of sera taken at all scheduled timepoints from the whole group. We also consider the extension of the search for anti-HLA Abs with utilization of Luminex Labscreen method which enables to calculate the mean fluorescence intensity and thus to assess the concentration of detected antibodies.

## Figures and Tables

**Table 1:** Patients characteristics (*n* = 30).

Median age (range)	
Recipient	37 (13–57) years
Donor	36 (19–55) years
Mean time from diagnosis to allo-HSCT (range)	0.75 (0.63–10.3) years

	Number (%)

Sex	
Donor	
Male	19 (63.3%)
Female	11 (36.7%)
Recipient	
Male	16 (53.3%)
Female	14 (46.7%)
Sex matching	
Male donor, male recipient	10 (33.3%)
Female donor, female recipient	5 (16.6%)
Male donor female recipient	9 (30%)
Female donor, male recipient	6 (20%)
HLA- mismatch	
Antigen A	4 (13.3%)
Antigen C	12 (40%)
Antigen DQ	2 (6.6%)
Allele A	2 (6.6%)
Allele B	5 (16.6%)
Allele DQ	3 (10%)
Antigen B + Antigen C	1 (3.3%)
Antigen A + Allele B	1 (3.3%)
Primary indication for allo-HSCT	
Acute lymphoblastic leukemia (ALL)	6 (20%)
Acute myeloid leukemia (AML)	15 (20%)
Chronic myeloid leukemia (CML)	5 (16.6%)
Chronic lymphocytic leukemia (CLL)	1 (3.3)
Severe aplastic anemia (SAA)	2 (6.6%)
Paroxysmal nocturnal hemoglobinuria (PNH)	1 (3.35)
Preparative regimen	
Cyclophosphamide	1 (3.3%)
TBI + Cyclophosphamide	6 (20%)
TBI + Fludarabine	1 (3.3%)
Treosulfan + Fludarabine	6 (20%)
Busulfan + Cyclophosphamide	12 (40%)
Busulfan + Fludarabine	1 (3.3%)
Treosulfan + Cyclophosphamide	1 (3.3%)
Busulfan + Cyclophosphamide + Gemtuzumab Ozogamycin	1 (3.3%)
Rituximab + Alemtuzumab + Melphalan	1 (3.3%)
Immunosuppressive treatment	
Glycocorticoid	27 (90%)
Cyclosporine	30 (100%)
Mycophenolate mofetil	7 (23%)
Tacrolimus	1 (3.3%)
Source of cells	
Bone marrow	9 (30%)
Peripheral blood	21 (70%)

**Table 2:** Anti-HLA antibodies detected before and 30 days, 100 days and 1 year after allo-HSCT in 30 recipients.

No	Typing of mismatched HLA			Detected anti-HLA Abs with regard to allo-HSCT			
	Recipient	Donor	HLA-mismatch	Before	+30 days	+100 days	+1 year
1	C 0401	C 12XX	Antigen C	Many specificities	Not tested	DR15	Not tested
	C 0501	C 0501					
2	DQB1 0202	DQB1 0202	Allele DQB1	Many specificities	Not tested	B70	Not tested
	DQB1 0302	DQB1 0301					
3	C 0802	C 0802	Antigen C	Many specificities	Not tested	Not tested	Not detected
	C 1203	C 0303					
4	C 0501	C 0201	Antigen C	Not detected	B65, B46, B37, C36, C10	DRB1*15, DQB1*06	Not detected
	C 0702	C 0702					
5	A 0201	A 0201	Allele A	Not detected	Not detected	Not tested	C14, B62, C9, A26
	A 0302	A 0301					
6	C 0401	C 0401	Antigen C	Not detected	Not tested	Many specificities	Not detected
	C 1602	C 1502					
7	C 0802	C 0702	Antigen C	Not detected	Not tested	Many specificities	Not detected
	C 1502	C 0702					
8	DQB1 0202	DQB1 0303	Antigen DQB1	Not detected	Not tested	Not detected	Not detected
	DQB1 0301	DQB1 0301					
9	C 0303	C 0403	Antigen C	Not detected	Not tested	DR13	B82, B49
	C 0102	C 0102					
10	C 0303 C 0401	C 0401 —	Antigen C	A23, A24, B27, B35, B38, B40, C0803, C0804	Not tested	B45, A66	DQ8, DR4
11	C 0303	C 0403	Antigen C	B75, B46, DR13, DQ3	Not tested	Not detected	Not detected
	C 0602	C 0602					
12	DQB1 0301	DQB1 0301	Allele DQB1	Not detected	Not tested	Not detected	B82, A34, DQ8, DR4
	DQB1 0504	DQB1 0501					
13	C 0202	C 0202	Antigen C	Not detected	Not tested	Not tested	B70
	C 0102	C 0202					
14	DQB1 0602	DQB1 0602	Allele DQB1	Many specificities	Many specificities	Not detected	Not detected
	DQB1 0602	DQB1 0603					
15	C 0102 C 0602	C 0302 C 0602	Antigen C	Not detected	C18, DRB3*, DPB1*05, DRB104, DQB1*06	Not tested	Not tested
16	A 1101	A 24xx	Antigen A	Not detected	Not detected	Not tested	DQ2, DQ4, DQA02, DQA04
	A 2601	A 2601					
17	A 0205	A 0201	Allele A	DQB1*03, DRB1*04	Not detected	Not tested	Not tested
	A 2402	A 2402					
18	B 4102	B 4102	Antigen B, antigen C	Not detected	Not detected	Not detected	Not tested
	B 5601	B 5501					
	C 0401	C 0301					
	C 1703	C 1703					
19	A 2601	A 0201	Antigen A	DQ8, DR4	DQ8, DR4	Not detected	B45, A66, DR10, DR12
	A 3201	A 3201					
20	DQB1 0301	DQB1 0301	Antigen DQB1	A2, 2C A0302, B67	A2, C2, B67	A2, DR 16	Not tested
	DQB1 0302	DQB1 0402					
21	A 0201 A 2901	A 0205 A 2901	Allele A	B7, 7C, B60, B81, A2403, A2608, C0727, C0804	B7	B47, B63	Not tested
22	C 0501	C 0501	Antigen C	Not detected	Not detected	Not detected	Not detected
	C 1203	—					
23	C 0304 C 0702	— C 0702	Antigen C	A2 A0302, B2703, B3501, B3503, 4006, B4101, B45, B4604, B67, B76, B78, C0103, C0403, DR51, DR15, DQ6, DR16, DQA01 DPB39, DPB3901, DPB85, DPB8501, DQB0502	A2, A0207, A0302, B2703, B3503, B4006, B4101, B67, B76, C0403, DR51, DR15, DQ6, DR16 DPB39, DPB3901, DPB85, DPB8501, DQB0502	A2, DR51, DR16	DQ7, DQA05
24	B 3501	B 3503	Allele B	B42, A80, C17	Not detected	B77	Not detected
	B 5701	B 5701					
25	A 2402	A 03xx	Antigen A	Not detected	Not detected	Not detected	Not tested
	A 2601	2601					
26	B 3501	B 3501	Allele B	C7, DQ8	Not detected	Not detected	Not detected
	B 3502	B 35xx					
27	A 3001	A 01xx	Antigen A	DR10, DR11	B77, B38	Not detected	Not tested
	A 3101	—					
28	A 2501	A 2501	Antigen A	Not detected	B77, A36	Not detected	Not detected
	A 3201	A 23xx					
29	B 1801	B 1801	Allele B	A31	Not detected	B61, C15, B35	Not tested
	B 4402	B 4427					
30	B 3503 B 3501	B 3503 B 3503	Allele B	DQ5, DQ6, DQA01	C7, DQ6, DR51, DPB39, DPB3901, DPB85, DPB8501, DQB0502,	C7, DR 51, DPB14, DPB1401, DQB0502, DQB0602, DQB0608, DR0806, DR2, DQ6, DQA01	Not tested
